# The state-led approach to industrial heritage in China’s mega-events: capital accumulation, urban regeneration, and heritage preservation

**DOI:** 10.1186/s43238-024-00144-1

**Published:** 2024-08-07

**Authors:** Mengke Zhang

**Affiliations:** College of Humanities, EPFL, CM 2 267, Station 10, Lausanne, 1015 Switzerland

**Keywords:** Industrial heritage practices, Mega-events, State entrepreneurialism, Land transformation, Urban development

## Abstract

This paper employs a comparative analysis to investigate the state-led use of industrial heritage in major Chinese mega-events, delving into the three cases of the Guangzhou Asian Games, the Shanghai Expo, and the Beijing Winter Olympics. Examining the evolving practices led by Chinese governments reveals unique pathways for industrial heritage and showcases its diverse roles in economic development and societal transformation. The three cases illustrate the nuanced dynamics between market forces and state interventions, emphasising the importance of strategic planning and long-term considerations in mega-event-induced heritage practices. Mega-events serve as catalysts for urban regeneration, allowing governments to allocate substantial resources to conserve and repurpose industrial heritage. However, the current paper contends that the sustained benefits of industrial heritage hinge on thoughtful planning for long-term economic and social sustainability, emphasising the need to constrain a focus on short-term gains through land revenue. These reflections contribute to a nuanced understanding of the intricate interplay between heritage preservation, economic development, and sustainable urban planning in the context of China’s mega-events.

## Introduction

This paper investigates the intricate relationship between industrial heritage and mega-events in the context of China by employing a comparative analysis of the utilisation of industrial heritage across various instances. The main objective is to comprehensively understand past experiences and emerging trends in the state-led approach to industrial heritage concerning new phases in urban social development. Amidst rapid urban expansion and industrial restructuring over the past several decades, numerous industrial legacies have been left behind in both old cities and suburban areas. The heightened awareness of industrial heritage preservation, marked by the release of the 2006 Wuxi Protocol, signifies a significant shift at the national level. Lu et al. (2020) argue that the development path of China’s industrial heritage protection is influenced not only by international milestone documents embedded with Western values but also by China’s perception of its industrial development history and the prevailing socioeconomic and cultural environment. In particular, under the recent movements towards sustainable development and ecological civilisation, industrial heritage has evolved into a governmental tool to enhance urban economic value and preserve the uniqueness of local lifestyles.

This paper specifically focuses on the pivotal role of Chinese governments in shaping the discourse and practices of industrial heritage within the framework of mega-events. Chinese governments notably approach heritage practices through entrepreneurial strategies, considering heritage an important resource for urban development and redevelopment (Su 2015). These practices, which start from major Chinese cities and spread to lower-tier cities, unveil novel pathways for the use of industrial heritage and the potential for its symbiotic development with Chinese cities. Moreover, mega-events serve as potent catalysts for urban transformation, which on the one hand, empower the government to invest substantial resources in projects that concurrently promote the conservation and utilisation of industrial heritage. On the other hand, such mega-events leverage the label of industrial heritage to augment their cultural value.

Jones and Ponzini (2018) highlight a general gap in the literature on mega-events and urban heritage. While acknowledging the seeming contradiction between the pro-growth orientation of mega-events and the preservation-centric stance of heritage efforts, both domains confront analogous urban challenges across economic, infrastructural, tourism, and sociocultural dimensions. They argue that these shared concerns and disparities are inadequately addressed, particularly in terms of externalities and secondary effects, urban and political dynamics, mass tourism, and public expenditure (Jones and Ponzini 2018). Another major concern lies in the difference in research time frames within the two domains. Most studies on mega-events have short-term attention, predominantly focus on pre-events rather than post-events, while heritage practices unfold as prolonged endeavours that require an extended duration for understanding and evaluating their effects (Jones 2020).

This paper examines three scenarios of industrial heritage utilisation in mega-events within China: the 2010 Guangzhou Asian Games, the Expo 2010 Shanghai China, and the Olympic Winter Games Beijing 2022. While Guangzhou and Shanghai represent early efforts at industrial heritage preservation in mega-events, Beijing exemplifies recent innovations in industrial heritage practices. These three cases collectively demonstrate the evolving role of industrial heritage in mega-events and urban redevelopment. By integrating China’s mega-events and heritage studies, this paper aims to challenge existing frameworks and address the complex dynamics inherent in China’s urban development paradigm. It seeks to contribute to a more sustainable approach that reconciles the objectives of both mega-events and industrial heritage preservation.

Methodologically, this comparative research employs a combination of field observations and documentary analysis, covering historical documents, official reports, media coverage, and academic literature. Specifically, slightly different approaches to the three cases are adopted. Field observations were conducted in Guangzhou in February 2022 and Shanghai in May 2023, mainly through on-site visits to relevant locations and landmarks. These two mega-events occurred over a decade ago and have been extensively documented in the literature, particularly regarding land issues and local urban regeneration policies, which are the major focuses of this paper. The limitations related to these two case studies not having interviews are, however, acknowledged. In contrast, more extensive fieldwork was conducted for the Beijing case in December 2021, March and April 2022, and June 2023, including formal and informal interviews with stakeholders, architects, planners, local residents, and visitors. Given its recent occurrence and limited prior study, these first-hand materials increase the importance of the Beijing case in analysis and discussion, shedding light on recent trends in event-driven industrial heritage utilisation and its impact on urban redevelopment. Before delving into the three scenarios, this paper reviews the literature on mega-events and heritage in international and Chinese contexts.

## Mega-events, event-led regeneration, and uses of cultural heritage

Mega-events, by common definition, have a few major features, including a large volume of visitors, extensive mediated reach, high costs, and significant impacts on the built environment and the population (Müller 2015b). As a product of the globalisation era (Close 2010), mega-events can play an important role in a country’s economic and political status. Hosting cities and countries usually invest many resources in mega-event related constructions, expecting that mega-events can bring significant political and economic benefits to the city and region, such as catalysing economic restructuring and urban regeneration (Essex and Chalkley 2004; Garcia-Ramon and Albet 2000). Typically, for post-industrial cities, mega-event-related regeneration strategies serve to mobilise investment to create new city images and new employment opportunities (Gratton et al. 2005). Serving as more than a justification, leveraging mega-events could be a tool to attract more funding and political support to initiate, for instance, ‘event-themed’ regeneration (Smith 2014; Smith and Fox 2007). Kasimati (2003) summarises the potential benefits of hosting mega-events, including new event facilities and infrastructures, urban revival, and improvements in tourism, inward investment, international reputation, and public welfare, but also warned that its negative impacts may outweigh the benefits.

Since the early 2000s, developing countries in Asia, Africa, and Latin America have increasingly sought to host mega-events, aiming to apply the associated development model to globally market their cities. These efforts involve experimental mega-projects for urban regeneration and reterritorialisation, concentrating state resources for significant investments in sports and transportation infrastructure (Golubchikov 2017; Sánchez and Broudehoux 2013). However, such developments tend to be dominated by elites, prioritising and accelerating capital accumulation for the elite’s interests rather than benefiting ordinary people (Koch 2018). Commonly, the consequences of mega-events include an overpromising of benefits and an underestimation of costs, resulting in oversized construction and infrastructure development (Müller 2015a), damage and disruption to existing local communities (Sánchez and Broudehoux 2013), and environmental issues (Essex and Chalkley 2010). In response to these challenges, there has been a growing call for the sustainable construction of mega-events. For instance, in 2017, the International Olympic Committee (2017a, 2017b) released two important documents, namely, the Sustainability Strategy and the Legacy Strategic Approach, which stress the sustainable planning and utilisation of Olympic legacies. This has prompted discussions on finding a sustainable win‒win development model related to mega-events.

Recently, the relationship between mega-events and heritage has gained increasing attention. Hosting mega-events can significantly influence urban heritage, reshaping its understanding, definition, and utilisation. Several studies have highlighted positive interactions between mega-events and heritage, which catalyse innovative heritage renewal and promotion. Heritage can be mobilised through mega-events to change perceptions of the city and, for instance, generate post-industrial imaginaries (Tommarchi 2022; Tommarchi and Bianchini 2022). It can also serve as a cultural infrastructure to help support social cohesion and improve residents’ quality of life (Sanetra-Szeliga 2022). Therefore, there is a growing need to recognise the potential of exploiting heritage during mega-events for future urban development and enhancing heritage awareness (Purchla 2022). However, the nexus between mega-events and heritage can be a double-edged sword (Ponzini 2022). On the one hand, mega-events provide opportunities and resources to renovate heritage within accelerated timelines. Events can even serve as a mechanism to protect heritage sites from emergency situations (Jones 2017). On the other hand, focusing heritage solely on mega-event goals risks the commodification of heritage. Rapid transformation induced by mega-events may also cause stress on the collective memories of local communities (Simon and Braathen 2019).

Addressing mega-event and heritage-related issues requires long-term plans, goals, and integrated approaches. Derived from a European city-based research project, the Charter for Mega-Events in Heritage-Rich Cities (HOMEE 2021) is a pioneer in providing principles and recommendations to policymakers, event organisers, and heritage actors to leverage opportunities from mega-events while mitigating risks. The Charter emphasises four key themes: 1) consider and adapt to the characteristics of specific urban contexts; 2) align mega-event planning with long-term strategies; 3) advocate inclusive governance to bring together heritage and other actors; and 4) explore new heritage narratives while mitigating challenges and social and political conflict. This framework provides an initial foundation for enriching future research and practical experiences between mega-events and heritage beyond Europe in broader and more sophisticated contexts.

## Mega-event-driven accumulation and urban regeneration in China

In China, there is a pronounced enthusiasm for hosting mega-events embraced by major cities throughout the country. The impact of mega-events on a national scale and their symbolic significance in China were notably highlighted during the Beijing Summer Olympics in 2008 (Ren 2009). Previous research on China’s mega-events has explored various important research dimensions, emphasising the instrumental roles of mega-events in terms of capital mobilisation (Wu et al. 2016), showcasing soft power (Grix and Lee 2013), social engineering (Chong 2017) and controlling social behaviours (Broudehoux 2012). The multifaceted nature of China’s approach to mega-events extends beyond immediate economic and infrastructural considerations, encompassing the intricate interplay of urban economies, national identity and social dynamics.

Several studies have elucidated the complex dynamics of a mega-event urbanisation model in China, with the essence of mobilising all available resources to achieve large-scale land and financial leverage by central and local states (Zhao et al. 2017). Shin (2012, 2014) presents two facets of this mega-event spatial strategy; one emphasises urban expansion achieved through significant state investments in infrastructure, and the other focuses on the ‘spatial fix’ of hosting cities to facilitate further capital accumulation while minimising political conflicts. The key to realising capital accumulation is to expedite land sales, often through the process of relocating old state-owned industries and urban villages; thus, local government financing platforms (LGFPs) play a pivotal role in financialisation (Wu et al. 2016). This unique urbanisation boom is stimulated by the strong developmental imperative and soft budget constraints of mega-event organisers (i.e., local governments) (Bao et al. 2019). However, the mega-event-driven model has raised concerns about its long-term sustainability, as well as socioeconomic and spatial consequences, such as significant government debts (Wu et al. 2016; Zhao et al. 2017), displacement of work migrants and underclass populations, and spatial inequality between the urban centre and periphery (Lin et al. 2018).

This mega-event approach has a close link with China’s urban growth model, with a significant focus on land issues. Many scholars have highlighted that the lens of land has been crucial to understanding great urban transformation in China over the past few decades (Hsing 2010; Wu 2015). Since the land reform in the late 1990s, relying on the land market, local governments in China have developed a logic of ‘land finance’ or a land-driven growth model through real estate to obtain the large amount of capital needed for urban construction and expansion. This is considered a form of urban development with Chinese characteristics, defined by Wu (2018) as ‘state entrepreneurialism’, which combines planning centrality and market instruments. It emphasises the increasing power of the central state in recent years by fixing developmental objectives implemented by local governments and state bodies (Li et al. 2022). Despite regular evolution, the state maintains an overwhelming role in urban development and governance that penetrates everyday life. Furthermore, recent trends in China’s state-led urban development are noteworthy, including promoting more spatially holistic resource allocation and eco-friendly development under the concept of ecological civilisation (Marinelli 2018; Pow 2018; Ren 2012), as well as creating more complex consumption-based activities as driving forces for urban economic growth (Theurillat 2021; Theurillat and Graezer Bideau 2022). These approaches have had an increasing impact on China’s mega-event-driven development.

## The conservation, adaptive reuse, and commodification of industrial heritage in China

The preservation of China’s industrial heritage has been underway for approximately two decades. However, the experiences related to this practice are spatially and temporally unstable and reshaped by the rapidly evolving economic and social processes of both the past and the ongoing present. Since the 1980s, economic reforms have led to the transition from a planned to a market economy and left behind numerous urban industrial sites that were retreated from the economic sector (Hsing 2010; Wu 2007). Many abandoned industrial sites, which were originally designated as production spaces for urban workers, have become integral parts of urban centres due to rapid urban expansion over the past four decades. Many of them were already demolished and redeveloped before the awareness of industrial heritage conservation in China emerged (Qian 2023). The Nizhny Tagil Charter, released by The International Committee for the Conservation of Industrial Heritage (TICCIH) in 2003, along with subsequent movements at the international level, had a profound influence on China’s understanding and definition of industrial heritage. In 2006, the release of the Wuxi Proposal by the State Administration of Cultural Heritage (SACH) indicated a nationwide advocacy of industrial heritage conservation while maintaining a nuanced difference from the international context, which is linked to external factors such as urban planning, social amenities, and market demands in China’s specific social context (Lu et al. 2020).

Adaptive reuse, which is an internationally accepted strategy for effectively retaining industrial heritage, has been widely used in China to recognise its substantial industrial legacies in the post-reform era (Chen and Judd 2021). Multiple forms of adaptive reuse have been identified. Yang et al. (2019) categorise three types of reuse models: 1) the industrial heritage museum model, which is often led by the government and nonprofit-driven but limited to a relatively small scale; 2) the tourist attraction development model, which is more profitable and involves business investment and catering to tourists’ interests, such as restaurants, bars, cafes and hotels; and 3) the creative park model, which transforms industrial remains into postmodern art spaces or offices. The latter originally adopted a bottom-up pattern within creative communities (e.g., artists, musicians, and architects) because of low rent in abandoned industrial spaces but later became more government organised under a culture-led spatial strategy (Gu 2014; Lu et al. 2020). Recently, scholars have observed that China’s industrial heritage practices have become more diverse and sophisticated with a mixture of different models for exploring the possibility of enhancing economic rewards while sustaining social and cultural capital (Qian 2023).

A common argument is that state entrepreneurialism triggers the commodification of urban heritage in pursuit of maximising economic benefits (Su 2015). Within the context of rapid urban development in China, the discourses and practices related to industrial heritage undergo frequent reshaping due to the dual interests of governments and developers. The objectives of urban development, primarily based on ideologies of modernity and aimed at enhancing the built environment, are, however, often contradictory to the conservation of industrial heritage. Industrial heritage often acts as an instrument and raw material for new development, such as by applying the concept of culture-led regeneration to relieve the tension between conservation and regeneration (Lu et al. 2020).

Many studies have shown a complex decision-making process for industrial heritage practices intertwined with state-market-society relations (Liang and Wang 2020; Yang and Qian 2023; Yang et al. 2019). In addition to the state-capital coalition, a few parties directly engage in the process, including local governments, the banking system, state-owned enterprises (SOEs) or private developers, and design consultants, while heritage specialists, ordinary urban residents and local communities are often conspicuously absent (Chen and Judd 2021). The role of the government is crucial in setting the direction of development and making early investments. Then, capital investors (SOEs or private investors) gain dominance in the planning and redevelopment process of industrial heritage, which typically aligns with the profit-making imperatives of property development (Chen and Judd 2021). In many cases, heritage value is sacrificed for short-term profitability in property development. The ownership of land rights obtained by developers plays an important role and can even constrain government intervention, not to mention other parties (Yang et al. 2019). Therefore, existing partnerships between state and nonstate institutions need to be challenged, and industrial heritage culture deserves increasing consideration for the sustainability of conservation efforts (Qian 2023).

It is evident that both industrial heritage reuse and mega-events are anchored in China’s land-based urban development model but are simultaneously trapped by the constraints of this model. The marketised environment places industrial heritage at a low priority, often viewing it as a barrier to property development. Similarly, the fervour for capital accumulation through land revenues in mega-events can result in substantial debt accumulation and exacerbated spatial and social inequalities. In the following, three empirical cases are examined, with the aim of shedding light on the intricate interplay between hosting mega-events and the reuse of industrial heritage.

## Scenario 1: Guangzhou Asian Games and the Taigucang Wharf

The 2010 Asian Games in Guangzhou showcased the revival of Taigucang Wharf (太古仓码头), which served as the starting point for the opening ceremony cruising and hosted various related Asian Games activities. This event marked a significant reintroduction of the once-abandoned wharf into the public sphere, accompanied by a newly defined functional position.

Situated on the east bank of the back channel of the Pearl River in Guangzhou, Taigucang Wharf has a history dating back to the 1900s when it was originally constructed by the British firm Swire Pacific. At that time, it stood as the highest-volume wharf warehouse facilitating overseas trade to and from Guangzhou. Following the establishment of the People’s Republic of China, the wharf was taken over by the state-owned Guangzhou Port Authority, later becoming part of the Guangzhou Port Group. In 1965, it gained the status of a national first-tier port open to the outside world, experiencing frequent trade activities until the early stages of the reform and opening up period (Fan 2012). However, with the construction of a seaport in southern Guangzhou from the late 1990s to the early 2000s, the cargo throughput of the Pearl River gradually declined, necessitating the transformation of Taigucang Wharf.

Recognising the historical and cultural significance of Taigucang Wharf as a vital relic of Guangzhou’s modern foreign trade and port transportation, the Guangzhou Municipal Government made a decisive commitment to its preservation and renovation. In approximately 2007, the Guangzhou Port Group undertook a substantial investment, injecting 80 million RMB, and collaborated with private capital amounting to 100 million RMB for the transformation of the Taigucang Wharf area. The primary objective was to convert the wharf area into an ‘urban living room’, designed as a waterfront public activity space open to the public. It also included the introduction of diverse cultural and commercial businesses strategically aligned with market-oriented operations. The revitalisation project aimed not only to preserve the historical legacy of Taigucang but also to integrate the legacy into the urban fabric, fostering a dynamic and multifunctional space for both residents and visitors.

Taigucang represents a typical example of Guangzhou's redevelopment efforts under the ‘three olds’ transformation initiative, which refers to the transformation of ‘old urban areas, old factories, and old villages’ (三旧改造) (see, e.g., Chen et al. 2020; Yuan 2016). Officially heralded as a successful model within this redevelopment framework, Taigucang embodies the core principles of the ‘three olds’ transformation policy, which is a strategic initiative by Guangdong Province aimed at balancing land revenue and spatialised capital accumulation (He 2012). While the bid for the Asian Games effectively bolstered Guangzhou’s urban influence, the ensuing infrastructure development incurred substantial debt for the city government (Bao and Li 2012; Chen et al. 2020), compelling a concerted effort to generate revenue through land-related channels. Starting in 2009, the decoration project for the Asian Games, which was aimed at improving the appearance of urban Guangzhou, catalysed the rapid pace of the ‘three olds’ transformation, illustrating the city’s proactive approach to urban redevelopment amid the economic and infrastructural demands associated with hosting the international event.

Categorised as ‘old factory’ buildings, the warehouses at Taigucang have undergone extensive renovation to facilitate the complete conversion from industrial to commercial functions, aiming to maximise land rent surplus. In contrast to Western developed countries where urbanisation is largely complete, China’s rapid urbanisation phase considers these old factory sites to be valuable and scarce developable lands, resulting in their elevated market value. According to the ‘three olds’ transformation policy, 60% of the land revenue is allocated to the original owner (in this case, the state-owned enterprise Guangzhou Port). The landowner, acting as a rational actor, thus naturally prioritises the maximisation of its interests in line with this policy (Yuan 2016).

**Fig. 1:**
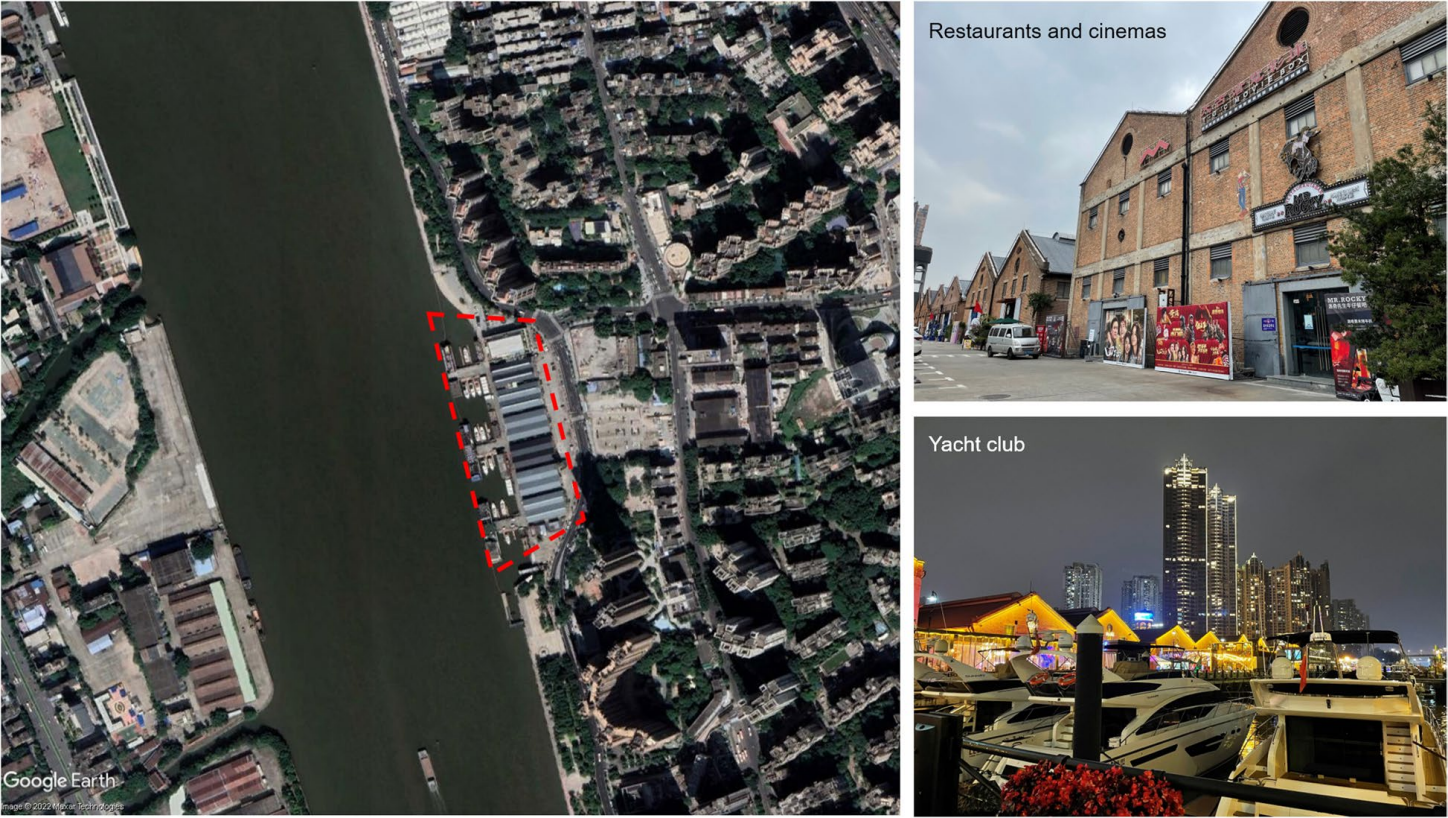
The renovated Taigucang Wharf in Guangzhou is surrounded by high-end communities (Source: map captured through Google Earth; photos taken by the author in February 2022)

The initial display of the renovated Taigucang in 2010 showcased a diverse array of functional uses, including a wine trade centre, special exhibition centre, cultural and creative fashion zones, clothing design studios, a yacht club, and various catering and entertainment facilities (Fan 2012; Lin 2009). However, my recent field observation in 2022, twelve years after the Taigucang transformation, revealed a notable shift. In contrast to the various cultural and creative industry zones that were initially established, all such zones have since been closed. Presently, the site predominantly features homogeneous Western-style restaurants, bars, live music venues, cinemas, and other purely commercial and entertainment-oriented establishments (Fig. 1). This transformation over the years reflects a discernible evolution in the functional makeup of Taigucang, raising questions about the sustainability and diversity of its initial cultural and creative endeavours.

Under market-oriented operations, the owner initially employed low rents to attract creative industries during the early stages of the transformation. However, as time passed and popularity increased, commercial enterprises, particularly those with stronger rent-paying abilities such as restaurants, gradually displaced cultural and creative industries with weaker commercialisation abilities. Consequently, the functions of Taigucang have undergone a profound transformation, with catering and entertainment now dominating, leading to a notable degree of functional homogenisation. Moreover, owing to its growing popularity, the second phase of development of Taigucang, namely, a pure commercial real estate project adjacent to those warehouses, was launched in 2022, with a total investment of more than 2 billion RMB (Li et al. 2024). This further demonstrates industrial heritage as a tool for developers to increase neighbouring land value for new property developments.

## Scenario 2: The Shanghai Expo and the Jiangnan Shipyard

In contrast to the relatively limited impact of the 2010 Asian Games on Guangzhou’s industrial heritage, the Expo 2010 Shanghai exerted a direct and profound influence on the spatial policy along the Huangpu River, stimulating large-scale industrial restructuring and facilitating population migration on both banks of the river. Throughout this process, the political narrative seamlessly integrated into the city’s long-term development plan, utilising the Shanghai Expo as a strategic tool to achieve the spatial fix of traditional industrial sites (Chan and Li 2017). This extensive transformation can be further characterised as a state-led city-branding initiative. Notably, the Huangpu River shoreline underwent a remarkable shift from a productive to a public leisure function, now serving as a vibrant waterfront public open space (Ding and Wu 2020).

During the Expo-oriented restructuring, the Bureau of Shanghai World Expo Coordination played a pivotal role as a ‘super developer’ (Deng et al. 2016), actively engaging in the entire process of the relocation of industrial areas, land formation, and the renovation and reuse of industrial heritage along the Huangpu River. The government issued policies to coordinate various stakeholders, particularly to satisfy the interests of SOEs, as the factories on the original site all belonged to SOEs. This strategic alignment aimed to achieve capital accumulation through the land formation process, supporting subsequent development in both the Expo and post-Expo eras (Li and Xiao 2022).

**Fig. 2:**
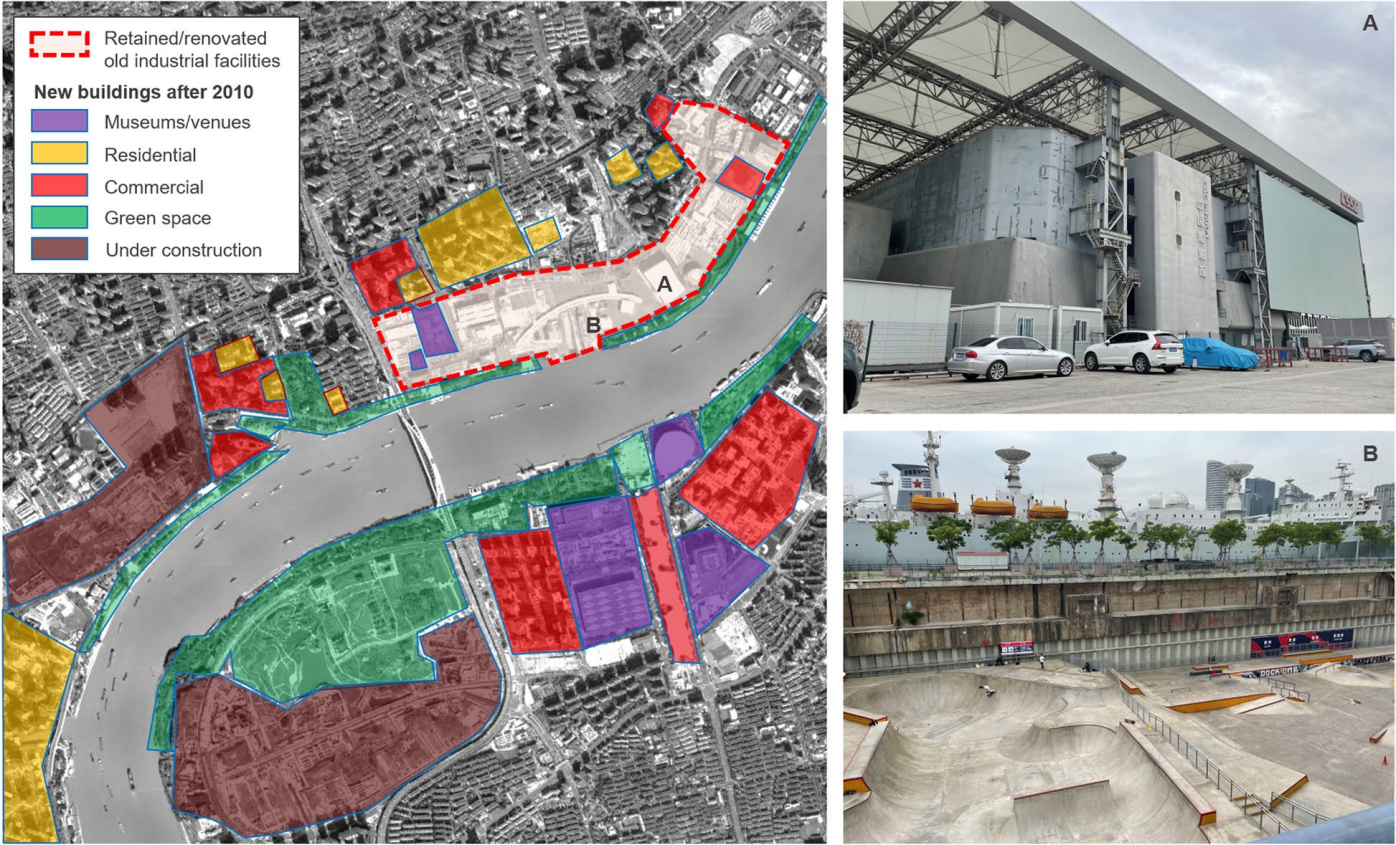
The renovation and demolition of industrial sites were driven by the 2010 Shanghai Expo, with industrial facilities being retained mostly on the west bank of the Huangpu River. A: The China Shipping Pavilion (CSSC Pavilion), once a workshop of the Jiangnan Shipyard, was renovated as an Expo venue and has served as an exhibition area after the Expo; however, it is often underused. B: A decommissioned survey ship currently used for tours, and a new skateboarding venue remodelled from a former shipyard in 2021 (Source: map made by the author; photos taken by the author in May 2023)

The adaptive reuse of industrial sites, exemplified by the Jiangnan Shipyard, was a prominent feature in the construction of the Shanghai Expo pavilions (Fig. 2). A three-tier protection strategy was implemented for the industrial facilities in the area, encompassing protected, retained, and renovated buildings, while the remainder faced demolition (Lv and Qiu 2010; Zhang 2015; Zuo 2010). In line with the exhibition characteristics of the Expo, large-scale structures such as Jiangnan Shipyard, owing to their significant structural and spatial advantages, were well suited to meet the Expo’s high traffic demands. Consequently, these expansive buildings underwent transformation into theme pavilions, supporting facilities, cultural activity venues, and so forth.

However, concerning post-Expo usage, the functional application of industrial buildings with substantial structures appears to be somewhat constrained. After the Expo, a small portion of venue buildings converted from industrial heritage were preserved as Expo memories and mostly repurposed as museums, contributing to the development of the museum industry in Shanghai. Nevertheless, the singular functional attribute of these museums has not brought popularity to the surrounding area, indicating that the reuse process of these venues has not been smooth.

Another criticism related to the renovation and reuse of these industrial heritage buildings is the lack of overall planning of the environmental characteristics and heritage landscape of the industrial building area, which adopted selective preservation methods with obvious tendencies (Zhang 2015). Although the construction scale of the World Expo was large, the construction period was short. As a result, the selected industrial heritage primarily included large-span buildings such as factories and warehouses, with few other buildings such as offices or other affiliated facilities being preserved. This approach failed to provide a comprehensive representation of the historical value and landscape features of industrial heritage. For example, after the Expo, the shipyard retained only a few disconnected industrial structures, preserving very little historical information about the original site and memory of the factory area. Simultaneously, almost all industrial buildings were revamped with new materials, leaving little of the original industrial architectural features that should have remained. Moreover, the construction of the Expo site accelerated the process of renovation of the old area but did so at the expense of large-scale demolition and construction. The complex situation of the former site in the area was marked by the juxtaposition of large-scale factories, dangerous houses and residential buildings, and local inhabitants were required to relocate as a whole, while their voices were mostly silent in the renovation process (Zuo 2010).

Over the subsequent decade following the Expo, the ongoing process of land formation and development for capital accumulation persisted in the area. Upscale residential and commercial zones were progressively constructed, contributing to a rapid escalation in the land value of the site. Despite the transformation, the former industrial area along the Huangpu River is now predominantly showcased to the public as a green public space, while the once-evident traces and narratives of industrial relics have faded into obscurity.

## Scenario 3: The Beijing Winter Olympics and Shougang Park

Established in 1919 at the base of Shijing Mountain (Shijingshan) in the western suburbs of Beijing, Shougang stands as one of China’s earliest steel factories, carrying the history of China’s industrial evolution from the Republic of China through the early years of the People’s Republic of China to the post-reform period. The transformation of Shougang Park has been intricately intertwined with Beijing’s two bids for the Summer and Winter Olympics over the past two decades.

Following Beijing’s successful bid for the Summer Olympic Games in 2001, the government strategically realigned its urban development plan, advocating for the relocation of traditional industries to make way for advanced service sectors. This initiative aimed to enhance the capital’s ecological environment and restructure its economy. In response to this directive, Shougang systematically phased out its steel production lines in Shijingshan, relocating all operations to Caofeidian in Hebei Province during the first decade of the new millennium (Luo et al. 2019). However, after the overall relocation, the original Shijingshan site experienced degradation and was unsuitable for subsequent development. Consequently, the transformation of the original Shougang Park stagnated for a long time.

**Fig. 3:**
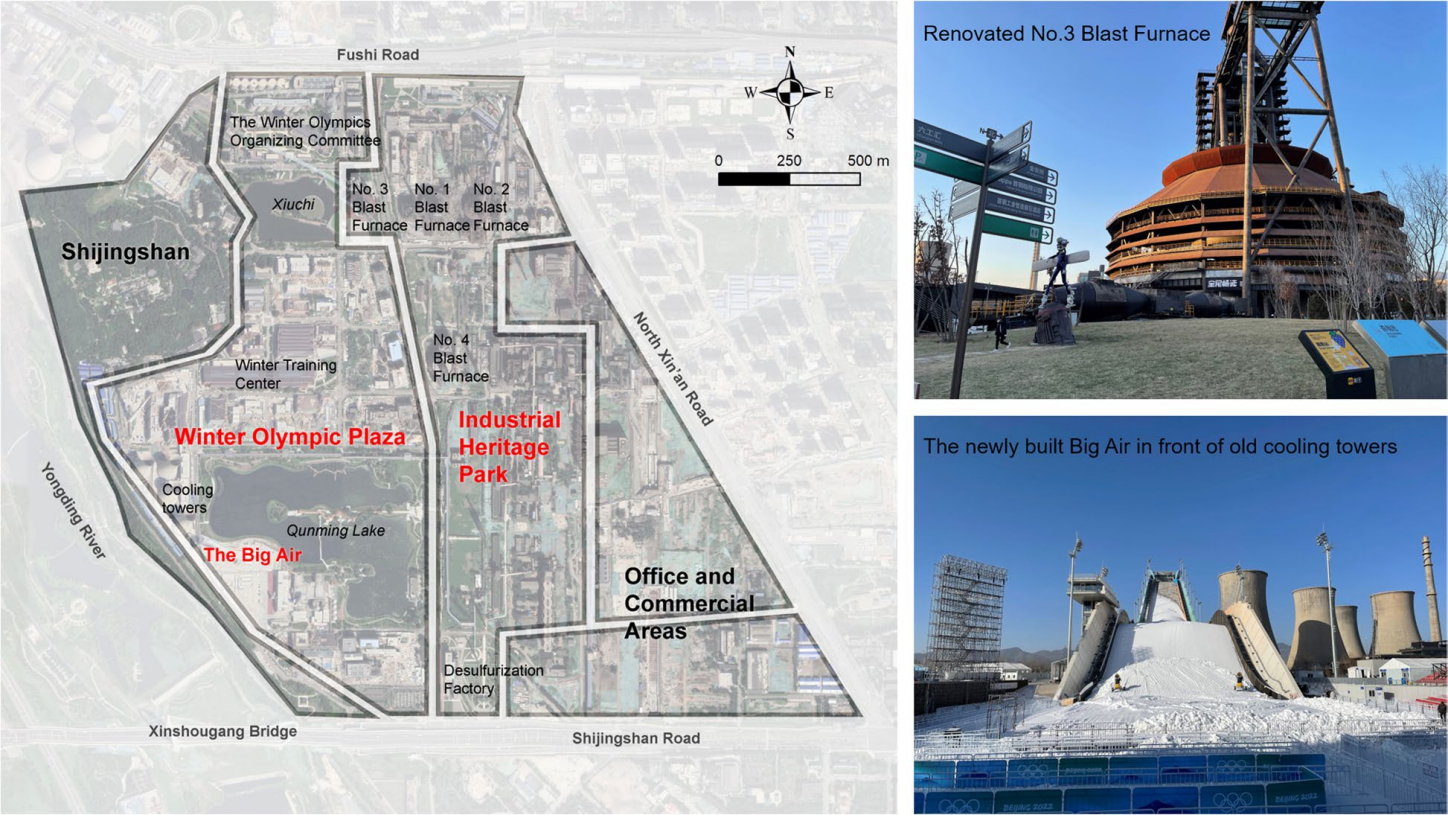
The renovated Beijing Shougang Park (Source: map made by the author; photos taken by the author in December 2021)

Only after the successful bid for the Beijing Winter Olympics in 2015 did Shougang Park enter a new phase of transformation and redevelopment. The Shougang Group negotiated with the Beijing Municipal Government and the Beijing Organizing Committee for the Olympic Games to establish the Big Air ski jumping venue within Shougang Park, the only snow sports competition venue in Beijing’s urban area (Deng et al. 2020). After the Winter Olympics, it became the world’s first permanently reserved big air venue (Fig. 3). Shougang also proactively allocated office space for the Beijing Organizing Committee for the Olympic Games, repurposing former silo buildings. Additional old factory workshops and warehouses underwent transformation to create the National Winter Training Center, serving as the training base for the national curling team, short track speed skating team, and figure skating team. Consequently, the winter sports industry has been integrated into Shougang Park, emerging as one of the pillar industries in the post-industrial era.

Building upon this foundation, numerous towering steel structures and industrial support facilities were preserved and repurposed into industrial-style commercial, entertainment, and office spaces, serving diverse functions such as shopping malls, upscale restaurants, bars, museums, and bookstores. This transformation also attracted renowned sports, e-sports, and sports media enterprises to establish their presence within the park. Simultaneously, Shougang Park opened extensive public leisure spaces and fostered a clean ecological environment along the riverside, contributing to increased popularity in the area.

Notably, the transformation and industrial restructuring of Shougang Park are inseparable from the key role played by the Shougang Group as a state-owned enterprise, which holds a very special status. Since the founding of the People’s Republic of China, the Shougang Group has shouldered significant industrial development tasks in different periods. With the deepening of economic reforms in the 1990s, the State Council granted the Shougang Group the right to establish investment projects, engage in capital financing, and exercise foreign trade autonomy (Luo et al. 2019). This granted the Shougang Group the ability to mobilise more resources and funds than ordinary local SOEs. In the 21st century, in response to the new Beijing urban development plan, Shougang underwent comprehensive relocation and made significant sacrifices. This relocation was a major project approved by the State Council, with both the State Council and the Beijing Municipal Government closely monitoring the utilisation of the original site after Shougang’s relocation. In this context, the Beijing Municipal Planning Department formulated a long-term plan and functional positioning to guide its transformation, proposing that ‘Shougang Park and its collaborative development area, serving as an important node in the western part of the city with the potential for upgrading and transformation, play a role in alleviating the functional concentration of the central city and improving the future functions of the city’ (Ju and Zhang 2021).

The Shougang Group took on responsibilities similar to those of a local government throughout the urban regeneration project. They managed the relocation and placement of industrial workers, coordinated overall planning and redevelopment of the original site, and oversaw long-term operations after the Olympics. With the objective of ensuring a stable and comprehensive process that includes planning, renovation, redesign, and post-Games operation and management, the implementation of this industrial park reuse strategy is expected to be effective in mitigating potential losses in the long run (Deng et al. 2020).

Taking the operational model of a shopping mall, namely, ‘Liu Gong Hui’ (六工汇), as an example, it can be observed that the Shougang Group’s approach to industrial park renovation prioritises long-term economic and social benefits. The land parcel hosting the shopping mall underwent a functional conversion from industrial to commercial under a government agreement, with the transaction land price significantly lower than that of the surrounding areas (Yu 2022). As part of the commitments in the agreement, the Shougang Group holds the long-term land use rights and is obligated to implement sustainable approaches to foster regional economic and social development in the western part of the city. Therefore, the Shougang Group established a joint venture operation company and enlisted an international professional urban renewal team to take charge of the specific operational planning. Notably, this joint operation company is entirely severed from land revenue, which is a deliberate measure to forestall rapid development and real estate sales solely for short-term profit motives (Yu 2022).

The Shougang Group has provided a clear vision for establishing a landmark within the revitalised old industrial zone, embodying a post-industrial cultural and sports creative hub. Positioned as one of the most recent and notable examples of industrial heritage reuse in China, Shougang Park aims to forge a new development path that embraces diverse functions and introduces an innovative operational model for the ongoing redevelopment of the industrial area. The project's future operational effectiveness is subject to ongoing evaluation as it progresses.

## Discussion and conclusion

The three cases of Guangzhou Taigucang Wharf, Shanghai Jiangnan Shipyard, and Beijing Shougang highlight the complex interplay between heritage preservation, economic development, and sustainable urban planning in the context of mega-events and industrial heritage reuse in major Chinese cities. Each city’s approaches and challenges provide valuable insights into the dynamics between the market and the state, which have shaped the post-industrial landscape in contemporary China. Table 1 provides a comparative overview of the three cases across various categories, illuminating their respective complexities and nuances.

**Table 1:** A comparison of three scenarios

Mega-events	2010 Guangzhou Asian Games	Expo 2010 Shanghai China	Olympic Winter Games Beijing 2022
Type of industrial heritage	Taigucang Wharf: wharf and warehouses	Shipyard, steel factory, warehouses	Shougang Park: steel factory
Scale	Small (0.07 km^2^)	Large (5.28 km^2^)	Large (2.91 km^2^)
Major stakeholders	Guangzhou Port Group (SOE) and Guangzhou Municipal Government	Bureau of Shanghai World Expo Coordination, Shanghai Municipal Government	Shougang Group (SOE), Beijing Organizing Committee for the Olympic Games, Beijing Municipal Government
Development approach	Market-driven with growing concerns	State-led city branding and public leisure	Dual Olympic influences, long-term consideration
Urban planning dynamics	SOE’s dominance on gated community regeneration	Integration of political narratives, large-scale transformation	SOE-government co-planning for subcentre development
Heritage preservation	Simple maintenance on buildings’ exterior	Selective preservation method, large-scale demolition	Comprehensive maintenance
Post-event functionality	Overemphasis on commercial, cultural and creative zones discontinued	Limited post-Expo functionality of preserved industrial buildings	Diverse functions integrated, needs further scrutiny

From a chronological perspective, the cases of Guangzhou and Shanghai represent initial forays and experiments in utilising industrial heritage during mega-events in China. These heritage practices from more than a decade ago successfully mobilised resources through mega-events but also revealed common pitfalls resulting from rapid transitions within this context. In the case of Taigucang Wharf, which was driven by the Guangzhou Asian Games and the ‘three olds’ transformation policy, the industrial heritage transformation leaned towards excessive commercialisation. The state-owned developer prioritised economic gains, leading to high rents that forced out cultural and creative industries. This resulted in a homogenisation of commercial activities and a focus on profit-driven property development. The transformation of industrial heritage along the Huangpu River in Shanghai adopted a short-term strategy focused on the Expo exhibition. Consequently, only large buildings suitable for conversion into exhibition halls were retained, while the narrative of the area's overall industrial heritage was lost. However, after the Expo ended, the utilisation of these large industrial buildings was severely limited, with many remaining vacant or underused as museums and exhibition halls.

Reflecting on the past decade of development in Guangzhou and Shanghai, the land issue is at the pivotal nexus between heritage preservation and economic development throughout both the transformation and post-event periods. Guangzhou adopted a market-driven model prioritising efficient land use, closely tied to rapid urbanisation and fostering land commodification. This emphasis on market-oriented approaches prioritised land rent surplus, favouring profit-driven endeavours over heritage conservation. Shanghai pursued a pronounced state-led strategy, which drove extensive restructuring but often did so at the expense of existing communities through widespread demolition and land transformations. Ultimately, both places fell into a land-driven growth model, propelled by the land market and diverging from the initial intent of heritage preservation.

In comparison, Beijing Shougang Park has adopted a more cautious development strategy for managing the relationship between mega-events and heritage, revealing a shifting approach to industrial heritage in China. Following the relocation of industrial production lines for the 2008 Summer Olympics, Shougang Park faced challenges and experienced a period of deterioration. Only after the successful bid for the 2022 Winter Olympics did a holistic strategy emerge for the reuse of industrial heritage driven by this opportunity, which has raised questions about its long-term effectiveness beyond serving the mega-event itself. While heritage was largely commodified or demolished in Guangzhou and Shanghai, Beijing has pursued more comprehensive maintenance as a political showcase, albeit incurring very high costs for preserving the heritage itself.

To explore more stable long-term redevelopment strategies, Beijing Shougang Park has attempted to make improvements in at least three aspects. The first aspect is the long-term stability of the redevelopment entity and its prominent position in heritage preservation strategies. While the Guangzhou developer prioritised land value enhancement, and the Shanghai case was spearheaded by the Bureau of Shanghai World Expo Coordination as a ‘super developer’ with a primary goal of achieving Expo objectives, the redevelopment of Beijing Shougang Park, although directly involving the Organizing Committee for the Olympics, has consistently been led by the Shougang Group. As a state-owned enterprise with a unique status, the Shougang Group operates like a small government, playing a pivotal role in safeguarding industrial heritage characteristics and driving the development of the western part of Beijing through the transformation of Shougang Park.

Second, the transformation efforts of Shougang Park align the objectives of the Winter Olympics and regional development with long-term planning. To achieve this, it prioritises long-term social benefits over short-term land revenue. The deliberate reduction of land prices by the government, as part of a strategic agreement with the Shougang Group, serves as a regulatory mechanism to mitigate the significant short-term costs of transformation and facilitate the realisation of long-term goals. Meanwhile, the worker community affiliated with the steel plant has been preserved to accommodate original workers. In addition, the entire industrial structure facilities that serve as collective memories of the past have also been retained.

Third, the renovated Shougang Park is centred on long-term operation after the Winter Olympics. It has introduced a professional commercial operation team with international experience, separating it from land profits to focus on long-term commercial operation benefits. Departing from the past mainstream development model focused on developing cultural and creative industries and museum visits, it creates a diverse functional integration driven by commercial and office uses as the core. In addition to developing businesses, more attention is given to incubating emerging high-tech industries, such as the industrial internet and virtual reality, complemented by local government subsidies to attract enterprises to settle in Shougang Park offices. This serves as the industrial foundation for its long-term development strategy.

These diverse industrial heritage practices, shaped by distinct land policies, government-enterprise dynamics, urban planning, and economic objectives, reflect Beijing Shougang Park's adoption of a more long-term holistic planning and heritage strategy. It represents a new exploration path for the utilisation of industrial heritage in mega-events, with its long-term effectiveness awaiting future assessment. Through comparative empirical analysis of the three cases, it is apparent that these events serve as catalysts, significantly shaping transformation processes and influencing local government decisions. Despite the temporary resource boost brought by mega-events, their impact is short-lived, concluding with the end of the event. Hence, the central argument posits that sustained benefits from industrial heritage require thoughtful planning for long-term economic and social sustainability, with a necessary constraint on land revenues. The case of Shougang underscores the importance of comprehensive and long-term planning to maximise industrial heritage value, integrating diverse functions. Economically, industrial heritage plays an important role in promoting regional development. Societally, it is displayed and reused in various cultural and technological forms, promoting new consumption practices and sociocultural lifestyles. Shougang Park's transformation revolves around core concepts such as heritage reuse, new technologies, ecology, environmental protection, tourism, and leisure, which also proposes a new development model for Chinese cities to help foster sustainable urban social and economic development through industrial heritage utilisation.

These recent heritage practices in China bear resemblances to the guidelines outlined in the Charter for Mega-Events in Heritage-Rich Cities (HOMEE 2021), which originated from a European context, particularly in advocating for long-termism and integrated approaches. They also emphasise the unique Chinese context in dealing with the intersections between mega-events and heritage preservation, notably the significant impact of land as a key element on both mega-event and heritage practices. These experiences provide valuable lessons for China’s future exploration of the effective utilisation of industrial heritage and long-term sustainable economic and social development. Moreover, they contribute to a deeper understanding of the relationship between mega-events and heritage, enriching the formation of a general theoretical framework with Chinese experiences. Future research could delve into comparative studies between China and other countries to further enrich the discourse on mega-events and industrial heritage, exploring variations in practices, challenges, and successes across different contexts. Additionally, longitudinal studies tracking the long-term impacts of event-oriented industrial heritage preservation and urban development could provide valuable insights for policymakers and practitioners in planning and decision-making processes.

## Data Availability

Not applicable.
